# HDL cholesterol efflux capacity and lipid profile in patients with systemic sclerosis

**DOI:** 10.1186/s13075-021-02443-9

**Published:** 2021-02-23

**Authors:** Iván Ferraz-Amaro, Esmeralda Delgado-Frías, Vanesa Hernández-Hernández, Hiurma Sánchez-Pérez, Laura de Armas-Rillo, Estefanía Armas-González, José David Machado, Federico Diaz-González

**Affiliations:** 1 Servicio de Reumatología, Hospital Universitario de Canarias, C/Ofra s/n, 38320 Santa Cruz de Tenerife, Spain; 2 Universidad Europea de Canarias, La Orotava, Tenerife, Spain; 3 Departamento de Farmacología, Facultad de Medicina, Universidad de La Laguna, Tenerife, Spain; 4 Departamento de Medicina Interna, Facultad de Medicina, Universidad de La Laguna, La Laguna, Spain

**Keywords:** Systemic sclerosis, Cholesterol efflux capacity, Modified Rodnan skin score

## Abstract

**Objective:**

It is well established that patients with systemic sclerosis (SSc) have a disrupted lipid profile and an increased cardiovascular risk. Cholesterol efflux capacity (CEC), the ability of high-density lipoprotein (HDL)-cholesterol to accept cholesterol from macrophages, has been linked to cardiovascular events. The aim of this study was to establish whether CEC and lipid profile were impaired in SSc patients with respect to controls and whether these changes were associated with disease-related data.

**Methods:**

Cross-sectional study encompassed 188 individuals: 73 SSc patients and 115 controls. CEC, using an in vitro assay, and lipoprotein serum concentrations were assessed in patients and controls. A multivariable analysis was performed to study the differences in CEC between patients and controls, and if SSc-related data could explain such differences.

**Results:**

The multivariable analysis adjusted for demographic characteristics, cardiovascular risk factors, and lipid-related molecules showed that total cholesterol (beta coefficient: − 22 [95%CI – 37 to – 7], *p* = 0.004), triglycerides (beta coefficient: 24 [95%CI 2–47], *p* = 0.033), lipoprotein A (beta coefficient: 22 [95%CI 2–43], *p* = 0.033), and CEC (beta coefficient: – 6 [95%CI − 10 to – 2]%,*p* = 0.002) were significantly different between patients and controls. Skin thickness, as assessed by modified Rodnan skin score, was independently associated with a lower CEC (beta coefficient: – 0.21 [95%CI – 0.37 to – 0.05]%, *p* = 0.011) after multivariable adjustment.

**Conclusion:**

SSc patients show an abnormal lipid profile with respect to controls including CEC. Skin thickness is independent and inversely associated with CEC in SSc patients.

**Supplementary Information:**

The online version contains supplementary material available at 10.1186/s13075-021-02443-9.

## Introduction

The HDL particle has multiple potentially anti-atherogenic properties. Much of this beneficial effect is thought to be mediated by its participation in the removal of cholesterol from macrophages in atherosclerotic plaques during a well-known anti-atherogenic process [1] termed “cholesterol efflux capacity” (CEC) [2]. Evidence suggests that CEC has a strong inverse association with carotid intima-media thickness (cIMT), the likelihood of angiographic coronary artery disease, independent of HDL cholesterol levels [3], and with the incidence of cardiovascular (CV) events in a population-based cohort [4]. Regarding to rheumatic diseases, it has been demonstrated that CEC is disrupted in patients with rheumatoid arthritis (RA) and systemic lupus erythematosus (SLE) [5], a finding associated with an increased subclinical atherosclerosis in both diseases [6, 7].

Systemic sclerosis (SSc) is a chronic autoimmune disease characterized by endothelial dysfunction, microvascular damage, inflammation, impaired coagulation/fibrinolysis and increased tissue fibrosis [8], and enhanced cardiovascular risk [9]. SSc has been associated with a disrupted lipid profile. Limited cutaneous SSc has been linked to low high-density lipoprotein (HDL) and total cholesterol serum levels [10] compared to controls. In another report, encompassing both limited and diffuse SSc, patients exhibited low serum levels of HDL cholesterol and high total cholesterol [11]. In this report, the atherogenic ratio LDL (low-density lipoprotein):HDL was significantly higher among SSc patients versus controls [11]. Furthermore, lipoprotein A serum levels have been found to be higher in patients with SSc compared to controls [12]. The mechanisms responsible for these modifications of lipid profile and the latter’s relevance in the development of atherosclerosis in patients with SSc are still unresolved. Moreover, CEC has not been explored with respect to controls in this disease.

Considering all these facts, the main objective of our study was to evaluate whether patients with SSc have a deregulated lipid profile and, for the first time, whether CEC is disrupted in these patients compared to healthy individuals. We also sought to identify patient characteristics, including demographic, clinical, and analytical features that could explain such potential CEC disturbances.

## Methods

### Study participants

This was a cross-sectional study that included 73 patients with SSc and 115 age- and sex-matched controls. All were 18 years old or older and were already enrolled, having met the American College of Rheumatology criteria for the classification of SSc [13]. They had been diagnosed by rheumatologists and were periodically followed-up at rheumatology outpatient clinics of our institution. For inclusion in the present study, SSc disease duration needed to be ≥ 1 year. Since glucocorticoids are often used in the management of SSc, patients taking prednisone were not excluded. None of the patients had established cardiovascular disease. The controls were community-based and were recruited by general practitioners in primary health centers. Controls with any history of inflammatory rheumatic diseases were excluded, as well as those with a history of cardiovascular disease. Moreover, patients and controls were excluded if they had a history of cancer or any other chronic disease, evidence of active infection, or a glomerular filtration rate < 60 ml/min/1.73 m^2^. The study protocol was approved by the Institutional Review Committee at Hospital Universitario de Canarias and all subjects provided informed written consent (CARESHUC Study: 2016_86).

### Assessments and data collection

Surveys in SSc patients and controls were performed to assess CV risk factors and medication use. Subjects completed a questionnaire and underwent a physical examination to determine anthropometric measurements and blood pressure. Medical records were reviewed to ascertain specific diagnoses, medications, and comorbidities. Hypertension was defined as a systolic or a diastolic blood pressure higher than, respectively, 140 and 90 mmHg. Disease duration for SSc was defined as the time since the onset of the first SSc-related symptom other than Raynaud’s phenomenon. SSc subtypes, limited and diffuse, were determined according to the distribution of skin thickness. The modified Rodnan skin score (mRSS) skin score was used to assess skin thickening [14]. This score has been commonly used as an outcome measure in clinical trials. It rates the severity of these features from 0 (normal) to 3 (most severe) in 17 distinct areas of the body and shows an acceptable degree of intra-rater variability. Esophageal involvement was defined as any sign of dysmotility evident on manometry. Articular involvement was determined by clinical evidence of joint swelling, deformity, contractures, and tendon friction rubs or radiographic evidence of joint space narrowing or erosion. Interstitial lung disease (ILD) was defined as signs of fibrosis on radiograph, high-resolution computed tomography, or by abnormal pulmonary function tests.

### Lipids and cholesterol efflux assessments

Fasting serum samples were collected and frozen at − 80 °C until analysis of circulating lipids. Cholesterol, triglycerides, and HDL cholesterol were measured using the enzymatic colorimetric assay (Roche). Lipoprotein A and lipoproteins were assessed using a quantitative immunoturbidimetric assay (Roche). Cholesterol ranged from 0.08 to 20.7 mmol/l (intra-assay coefficient of variation of 0.3%); triglycerides ranged from 4 to 1.000 mg/dl (intra-assay coefficient of variation of 1.8%); and HDL cholesterol ranged from 3 to 120 mg/dl (intra-assay variation coefficient of 0.9%). The atherogenic index was calculated using the total cholesterol/HDL-C ratio according to the Castelli formula. LDL cholesterol was calculated using the Friedewald formula. A standard technique was used to measure high-sensitivity C-reactive protein (CRP).

Macrophage-specific CEC was measured in apolipoprotein B-depleted plasma from SSc patients and controls using BODIPY cholesterol-loaded J774 cell line as previously described [4]. The CEC was calculated as the amount of effluxed BODIPY cholesterol expressed as a fraction of the initial cell content of BODIPY cholesterol. Each assay was performed in triplicate, and when the percentage of variation of every sample was higher than 7%, the sample was reassessed.

### Statistical analysis

Demographic and clinical characteristics were compared between SSc patients and controls using *χ*^2^ tests for categorical variables or Student’s *t* test for continuous variables (data expressed as percentages and mean ± standard deviation, SD). For non-continuous variables, either a Mann-Whitney U test was performed, or a logarithmic transformation was made, and data were expressed as a median and interquartile range (IQR). Univariable linear regression analyses were performed to establish the relation of demographics, traditional cardiovascular risk factors, lipid profile, and SSc-related data with CEC. Differences between patients and controls regarding lipid profile including CEC were assessed through multivariable linear regression analysis. To this end, we performed 3 different linear regression analyses. First, differences in lipid profile and CEC were assessed through univariable linear regression analysis. Second, Model 1 was constructed using as confounding variables those variables with a statistical “*p*” value lower than 0.20 in the differences between patients and controls in traditional cardiovascular risk factors (waist circumference and statin intake). Third, Model 2 was constructed adjusting for variables of Model 1 *plus* rest of lipid molecules, other than the one that is compared, in which differences disclosed a *p* value < 0.20 in the previous Model 1. To avoid collinearity, variables derived from a formula were excluded from the regression models (LDL cholesterol, LDL:HDL ratio, non-HDL cholesterol, apoB:apoA1, and atherogenic index). Similarly, the relation between mRSS and the lipid profile was studied through multivariable lineal regression analysis assuming the same criteria for confounding variables and collinearity. All analyses used a 5% two-sided significance level and were performed using SPSS software, version 21 (IBM, Chicago, IL, USA). A *p* value < 0.05 was considered statistically significant.

## Results

### Demographic, laboratory, and disease-related data

A total of 188 participants, 73 patients with SSc (50 patients (68%) with limited form and 23 patients (32%) with diffuse form) and 115 age- and sex-matched controls, were included in this study. Demographic characteristics of patients and controls, as well as disease-related characteristics of the participants, are shown in Table 1. There were no differences between patients and controls regarding body mass index and the presence of hypertension, current smoking, diabetes, or obesity. Only waist circumference and current use of statins (34% vs. 11%, *p* = 0.000) were found to be higher in patients with SSc. Many differences were found in the lipid profiles between patients and controls. In this sense, cholesterol, HDL and LDL cholesterol, and apolipoprotein A1 were found to be lower in SSc patients. In contrast, triglycerides, lipoprotein A, the apoB:A1 ratio, and the atherogenic index revealed higher serum levels in SSc patients. The mean CEC of HDL was significantly lower in SSc patients compared to controls (17 ± 11 vs. 8 ± 3%, *p* = 0.000) when the univariable analysis was performed.

**Table 1:** Demographics of SSc patients and controls vis-à-vis comorbidities, analytical, and disease-related data

	Controls (***n*** = 115)	SSc patients (***n*** = 73)	***p***
Demographics			
Female, *n* (%)	107 (93)	68 (93)	0.98
Age, years	60 ± 6	59 ± 1	0.40
BMI, mg/cm^2^	28 ± 5	29 ± 6	0.38
Waist circumference, cm	91 ± 14	96 ± 14	**0.042**
Systolic pressure, mmHg	131 ± 15	133 ± 19	0.54
Diastolic pressure, mmHg	84 ± 53	80 ± 13	0.53
Comorbidities, *n*(%)			
Hypertension	40 (35)	27 (37)	0.76
Current smoking	19 (17)	14 (19)	0.61
Diabetes	7 (6)	8 (11)	0.22
BMI > 30	30 (26)	22 (30)	0.55
Statins	13 (11)	25 (34)	**0.000**
Analytical data			
CRP, mg/dl	1.00 (1.00–3.00)	2.35 (1.15–4.29)	0.96
Cholesterol, mg/dl	223 ± 38	198 ± 39	**0.000**
Triglycerides, mg/dl	104 ± 52	153 ± 83	**0.000**
HDL cholesterol, mg/dl	66 ± 18	49 ± 13	**0.000**
LDL cholesterol, mg/dl	136 ± 35	111 ± 45	**0.000**
LDL:HDL cholesterol ratio	2.23 ± 0.84	2.50 ± 1.62	0.23
Non-HDL cholesterol, mg/dl	157 ± 37	149 ± 41	0.24
Lipoprotein A, mg/dl	16 (9.32)	23 (12.65)	**0.020**
Apolipoprotein A1, mg/dl	197 ± 34	168 ± 32	**0.000**
Apolipoprotein B, mg/dl	102 ± 22	100 ± 26	0.67
Apo B:Apo A ratio	0.54 ± 0.16	0.63 ± 0.30	**0.024**
Atherogenic index	3.58 ± 0.98	4.37 ± 1.74	**0.003**
Cholesterol efflux capacity, %	17 ± 11	8 ± 3	**0.000**
Systemic sclerosis-related data, *n* (%)			
Disease duration, years		9 (4–15)	
Modified Rodnan skin score, units		3 (1–6)	
Raynaud phenomenon		62 (85)	
Digital ulcers		8 (11)	
Calcinosis		7 (10)	
Arthritis		15 (21)	
Gastric reflux		29 (40)	
Pathological esophageal manometry		40 (55)	
Interstitial lung disease		16 (22)	
Pulmonary hypertension		16 (22)	
Anti-centromere antibody positivity		47 (64)	
Anti-Scl70 antibody		10 (14)	
Treatments			
Current prednisone use		15 (21)	
Prednisone, mg/day		5 (5–10)	
DMARDs, *n* (%)		10 (14)	
Azathioprine		4 (5)	
Methotrexate		2 (3)	
Mycophenolate		2 (3)	
Hydroxychloroquine		2 (3)	

Data represent patient numbers (%), means ± SD or median (IQR) when data were not normally distributed

*BMI* body mass index; *CRP* C-reactive protein; *DMARD* disease-modifying anti-rheumatic drug; *HDL* high-density lipoprotein; *LDL* low-density lipoprotein

Disease duration was 9 (IQR 4–15) years in SSc patients. Disease durations for the limited and diffuse types were 12 (IQR 6–17) and 8 (IQR 4–15) (*p* = 0.13) years, respectively (see Supplementary Table 1). The mRSS score was 3 (IQR 1–6; minimum–maximum 0–27) for the entire study population (Table 1) and no significant differences were found (*p* = 0.12) when this score was analyzed independently for the limited (3, IQR 0–6) and diffuse (12, IQR 6–17) forms (see Supplementary Table 1). The presence of digital ulcers and calcinosis was reported, respectively, in 11% and 10% of the patients. At the time the study was conducted, about one fifth of patients (21%) were taking prednisone with a median dose of 5 [IQR 5–10] mg/day. Additionally, at the time of the study, 47 (64%) patients were found to be positive for anti-centromere, and 10 (14%) were positive for anti-Scl70. Disease-modifying anti-rheumatic drug (DMARD) use was reported in 14% of the patients, including 5% azathioprine, 3% hydroxychloroquine, and 3% methotrexate. Other features related to the disease are shown in Table 1.

### Multivariable analysis of the differences in lipid profiles between SSc patients and controls

**Fig. 1:**
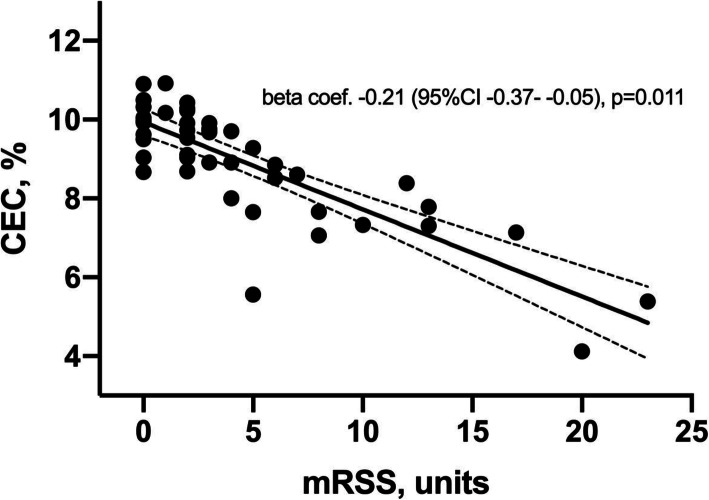
Multivariable analysis of the relation between modified Rodnan skin score (mRSS) and cholesterol efflux capacity (CEC)

Table 2 and Fig. 1 show the differences in lipid-related molecules between patients and controls. After adjusting for abdominal circumference and statins (Model 1 in Table 2), most of these molecules showed differences between the two populations. In this sense, total cholesterol, HDL and LDL cholesterol, apolipoprotein A1, and CEC were downregulated in SSc patients to a statistically significant degree. In contrast, triglycerides, lipoprotein A, the apolipoprotein B:A1 ratio, and the atherogenic index showed higher levels in SSc patients.

**Table 2:** Multivariable analysis of the differences in lipid profiles between SSc patients and controls

	Controls (***n*** = 115)	SSc patients (***n*** = 73)	Univariable model	Model 1	Model 2 beta coef. (95% CI), ***p***	
Lipid profile						
Cholesterol, mg/dl	223 ± 38	198 ± 39	**0.000**	**0.001**	− 22 (−37 to − 7)	**0.004**
Triglycerides, mg/dl	104 ± 52	153 ± 83	**0.000**	**0.001**	24 (2–47)	**0.033**
HDL cholesterol, mg/dl	66 ± 18	49 ± 13	**0.000**	**0.000**	− 1 (− 5 to 3)	0.75
LDL cholesterol, mg/dl	136 ± 35	111 ± 45	**0.000**	**0.001**		
LDL:HDL cholesterol ratio	2.23 ± 0.84	2.50 ± 1.62	0.23	0.098		
Non-HDL cholesterol, mg/dl	157 ± 37	149 ± 41	0.24	0.24		
Lipoprotein A, mg/dl	16 (9–32)	23 (12–65)	**0.020**	**0.055**	22 (2–43)	**0.033**
Apolipoprotein A1, mg/dl	197 ± 34	168 ± 32	**0.000**	**0.000**	− 9 (− 19 to 0)	0.062
Apolipoprotein B, mg/dl	102 ± 22	100 ± 26	0.67	0.52		
Apo B:Apo A ratio	0.54 ± 0.16	0.63 ± 0.30	**0.024**	**0.010**		
Atherogenic index	3.58 ± 0.98	4.37 ± 1.74	**0.003**	**0.001**		
Cholesterol efflux capacity, %	17 ± 11	8 ± 3	**0.000**	**0.000**	− 6 (−10 to − 2)	**0.002**

Data represent means ± standard deviation; Model 1: Adjusted for waist circumference and statins; Model 2: Adjusted for Model 1 + rest of lipid molecules (with a *p* value < 0.20 in the univariable analysis) other than the one that is compared. Because collinearity LDL cholesterol, LDL:HDL ratio, non-HDL cholesterol, apoB:apoA, and atherogenic index were excluded from the multivariable analyses in Model 2, *HDL* high-density lipoprotein; *LDL* low-density lipoprotein

Because lipid-related molecules are interrelated (they share metabolic pathways and distinguishing the effect of one from another is difficult), we performed a multivariable analysis adjusting for demographics and cardiovascular risk factors plus all the lipid-related molecules that were found to be different between patients and controls in Model 1 (Model 2 in Table 2). Because of collinearity, lipid molecules derived from a formula were excluded from the regression model (LDL cholesterol, LDL:HDL ratio, non-HDL cholesterol, apoB:apoA1, and atherogenic index). Total cholesterol (beta coef. − 22 [95%CI – 37 to − 7], *p* = 0.004), triglycerides (beta coef. 24 [95%CI 2–47], *p* = 0.033), and lipoprotein A (beta coef. 22 [95%CI 2–43], *p* = 0.033) maintained their differences between patients and controls. Interestingly, CEC conserved its downregulation in SSc patients after adjusting for other lipid profile-related molecules (beta coefficient − 6 [95%CI – 10 to − 2] %, *p* = 0.002). Remarkably, HDL cholesterol was not different between patients and controls after the multivariable analysis.

### Association of demographics, lipid profile, and disease-related data with efflux capacity in SSc patients and controls

Demographics and lipid profiles were, in general, not associated with CEC in either patients or controls. In this sense, only abdominal circumference showed a positive association with CEC in patients (beta coefficient 0.09 [95%CI 0.03–0.14], *p* = 0.002), though not in controls. Similarly, no traditional cardiovascular risk factors were linked to CEC in either population. Regarding lipid profiles, no correlations were identified between the standard lipid profile molecules and CEC. Remarkably, the use of statins was not related to CEC in patients or controls. Lastly, concerning SSc-related data, a negative association between mRSS and CEC was identified (beta coef. − 0.19 [95%CI − 0.34 to − 0.04]%, *p* = 0.014) (Table 3). Disease duration was not related to CEC in either the diffuse or limited SSc types. When patients with the diffuse form were divided according to the duration of the disease, those within the first 5 years of disease onset had a significantly higher CEC than those with 5 or more years of disease duration in the univariable analysis (14 ± 1 vs 9 ± 3, *p* = 0.035). However, these results should be taken with caution because of the 23 patients with diffuse form, only 3 had a disease duration lower than 5 years. Moreover, the use of calcium channel blockers, PDE5 inhibitors, or endothelin receptor antagonists was not related to CEC (data not shown).

**Table 3:** Relation of demographics, lipid profile, and disease-related data with cholesterol efflux capability in SSc patients and controls

	Cholesterol efflux capacity beta coef. (CI95%), ***p***	
	Controls (***n*** = 115)	Patients (***n*** = 73)
Age, years	− 0.03 (− 0.35 to 0.30), 0.88	− 0.04 (− 0.11 to 0.04), 0.32
Male	− 3.34 (− 11.34 to 4.66), 0.41	− 1.67 (− 4.81 to 1.47), 0.29
Body mass index, kg/m^2^	− 0.18 (− 0.59 to 0.24), 0.41	0.14 (0.00 to 0.28), 0.050
Abdominal circumference, cm	− 0.13 (− 0.27 to 0.02), 0.097	**0.09 (0.03 to 0.14), 0.002**
Systolic blood pressure, mmHg	− 0.14 (− 0.27 to 0.00), 0.057	0.01 (− 0.03 to 0.06), 0.57
Diastolic blood pressure, mmHg	− 0.02 (− 0.06 to 0.02), 0.39	0.01 (− 0.05 to 0.08), 0.67
Cardiovascular comorbidities		
Smoking	4.05 (− 1.39 to 9.50), 0.14	1.50 (− 0.47 to 3.47), 0.13
Diabetes	0.81 (− 7.72 to 9.35), 0.85	1.63 (− 0.87 to 4.12), 0.20
Hypertension	− 2.75 (− 7.00 to 1.51), 0.20	0.41 (− 1.25 to 2.07), 0.62
Statins	− 2.73 (− 9.16 to 3.69), 0.40	0.35 (− 1.32 to 2.02), 0.68
Analytical and lipid profiles		
CRP, mg/dl		
Cholesterol, mg/dl	0.04 (− 0.02 to 0.09), 0.17	0.01 (− 0.01 to 0.03), 0.35
Triglycerides, mg/dl	− 0.02 (− 0.06 to 0.03), 0.43	0.01 (− 0.01 to 0.02), 0.36
HDL cholesterol, mg/dl	0.08 (− 0.03 to 0.20), 0.16	− 0.00 (− 0.07 to 0.07), 0.97
LDL cholesterol, mg/dl	0.02 (− 0.04 to 0.08), 0.43	0.01 (− 0.01 to 0.03), 0.19
LDL:HDL cholesterol ratio	− 0.31 (− 2.78 to 2.17), 0.81	0.11 (− 0.42 to 0.64), 0.69
Non-HDL cholesterol, mg/dl	0.01 (− 0.04 to 0.07), 0.66	0.01 (− 0.01 to 0.03), 0.37
Lipoprotein A, mg/dl	0.04 (− 0.02 to 0.11), 0.16	0.01 (− 0.01 to 0.02), 0.41
Apolipoprotein A1, mg/dl	0.03 (− 0.03 to 0.09), 0.33	0.01 (− 0.02 to 0.03), 0.62
Apolipoprotein B, mg/dl	− 0.01 (− 0.10 to 0.09), 0.91	0.01 (− 0.02 to 0.04), 0.58
Apo B:Apo A ratio	− 5.19 (− 18.11 to 7.73), 0.43	− 0.39 (− 3.34 to 2.56), 0.79
Atherogenic index	− 0.52 (− 2.63 to 1.60), 0.63	0.01 (− 0.50 to 0.52), 0.97
SS- and treatment-related data		
*log* Disease duration, years		− 0.51 (− 1.47 to 0.45), 0.29
Modified Rodnan skin score, units		**− 0.19 (− 0.34 to − 0.04), 0.014**
Raynaud phenomenon		− 0.54 (− 2.89 to 1.80), 0.64
Digital ulcers		− 2.50 (− 4.99 to 0.01), 0.049
Calcinosis		− 0.53 (− 3.25 to 2.18), 0.70
Arthritis		0.28 (− 2.26 to 1.71), 0.78
Gastric reflux		0.87 (− 0.78 to 2.53), 0.30
Pathological esophageal manometry		0.15 (− 1.78 to 2.08), 0.88
Interstitial lung disease		− 1.34 (− 3.24 to 0.55), 0.16
Pulmonary hypertension		− 1.14 (− 3.15 to 0.86), 0.26
Anti-centromere antibody positivity		− 0.34 (− 2.08 to 1.40), 0.70
Anti-Scl70 antibody positivity		1.80 (− 0.49 to 4.09), 0.12
Current prednisone		− 0.69 (− 2.67 to 1.28), 0.49
*log* Prednisone		− 2.22 (− 6.83 to 2.40), 0.32
DMARDs		− 0-00 (− 2.33 to 2.33), 0.99
Azathioprine		− 0.33 (− 3.95 to 3.07), 0.80
Methotrexate, *n* (%)		0.96 (−3.93 to 5.86), 0.70
Hydroxychloroquine, *n* (%)		− 0.85 (− 5.74 to 4.05), 0.73

*ANA* antinuclear antibodies; *BMI* body mass index; *CRP* C-reactive protein; *DMARD* disease-modifying anti-rheumatic drug; *HDL* high-density lipoprotein; *LDL* low-density lipoprotein

### Modified Rodnan skin score’s association with lipid profile molecules and CEC

mRSS was positively related to presence of hypertension, but negatively associated with CEC when the univariable correlations were assessed (Table 4). When the relation of mRSS to these lipid-related molecules was adjusted for traditional CV risk factors, similar results were found. Moreover, when the relation between mRSS and CEC was additionally adjusted for other lipid-related molecules, its significance was conserved (beta coef. − 0.21 [95%CI − 0.37 to − 0.05]%, *p* = 0.011) (Fig. 1). When CEC was analyzed by SSc forms, a significant difference was found between the limited and diffuse forms (7.98 ± 3.33 vs 9.79 ± 3.52, *p* = 0.035). However, when this difference was analyzed by adjusting for demographic characteristics and cardiovascular comorbidities, no significant difference was found in CEC between the two forms of SSc (*p* = 0.18) (see Supplementary Table 1).

**Table 4:** Modified Rodnan skin score association with lipid profile molecules and CV risk factors in SSc patients

	Modified Rodnan skin score			
	Pearson’s r	p	Model 1 Beta coef. (95% CI), ***p***	Model 2 Beta coef. (95% CI), ***p***
Age, years	0.202	0.13		
Male	− 0.142	0.29		
Body mass index, kg/m^2^	− 0.028	0.84		
Abdominal circumference, cm	−0.018	0.90		
Systolic blood pressure, mmHg	0.016	0.91		
Diastolic blood pressure, mmHg	− 0.174	0.20		
Cardiovascular comorbidities				
Smoking	0.009	0.94		
Diabetes	0.182	0.17		
Hypertension	**0.294**	**0.025**		
Statins	0.109	0.41		
Analytical and lipid profiles				
Cholesterol, mg/dl	− 0.176	0.25	− 1 (−3 to 1), 0.25	−0.29 (−1.36 to 0.78), 0.59
Triglycerides, mg/dl	0.150	0.33		
HDL cholesterol, mg/dl	0.031	0.84		
LDL cholesterol, mg/dl	− 0.237	0.10	− 2 (− 4 to 0), 0.10	
LDL:HDL cholesterol ratio	− 0.191	0.19	− 0.03 (− 0.08 to 0.02), 0.19	
Non-HDL cholesterol, mg/dl	− 0.182	0.23	− 1 (− 3 to 1), 0.23	
Lipoprotein A, mg/dl	0.072	0.64		
Apolipoprotein A1, mg/dl	0.168	0.27	0.61 (− 0.80 to 2.03), 0.39	
Apolipoprotein B, mg/dl	− 0.220	0.15	− 0.91 (− 2.14 to 0.33), 0.15	− 0.04 (− 0.85 to 0.78), 0.93
Apo B:Apo A ratio	− 0.280	0.062	− 0.00 (− 0.02 to 0.00), 0.062	
Atherogenic index	− 0.100	0.51		
Cholesterol efflux capacity, %	**− 0.323**	**0.014**	**− 0.19 (− 0.34 to − 0.04), 0.014**	**− 0.21 (− 0.37 to − 0.05), 0.011**

mRSS is considered the independent variable in the multivariable analyses. Model 1: adjusted for gender, age, and hypertension; Model 2: adjusted for Model 1 + rest of lipid molecules (with a *p* value < 0.20 in the univariable analysis) other than the one that is compared. Because collinearity parameters derived from a formula (LDL cholesterol, LDL:HDL ratio, non-HDL cholesterol, apoB:apoA, and atherogenic index) were excluded from the multivariable analyses in Model 2

*HDL* high-density lipoprotein; *LDL* low-density lipoprotein

## Discussion

In the current report, we analyzed the lipid profile and for the first time the CEC in a series of SSc patients with respect to controls. Based on our results, SSc patients show a dysregulated lipid profile and a downregulated CEC, the former was independent of traditional CV risk factors, statin use, or other variations in the lipid profiles. Remarkably, mRSS, a marker of disease severity and damage, is inversely related to CEC in patients with SSc.

Although few studies address this subject, previous reports have described SSc as having a disrupted lipid profile. However, the trend for this disturbance has been contradictory depending on the series studied. Our finding of higher lipoprotein A serum levels is in accordance with a previous study on this molecule in SSc patients [12]. In addition, the fact that SSc display lower levels of total cholesterol and higher triglycerides than controls has also been previously described [10, 11], although in smaller cohorts that lacked multivariable analysis. The lipid profile differences between patients and controls found in our study are in accordance with the “lipid paradox” [15] described in other inflammatory diseases like RA [6, 16, 17] or SLE [18]. This means that untreated inflammatory diseases are associated with lower levels of total cholesterol and LDL cholesterol, and it is believed that this may stem from the lipid-lowering effects of systemic inflammation. To date, our study is the largest one in which the lipid profile was studied in patients with SSc. In addition, our sample size permitted multivariable analysis, and our study included a control population. For these reasons, we believe that our findings regarding dyslipidemia in SSc patients can unlikely be attributed to confounding factors.

SSc patients suffer a greater risk of CV diseases than controls [9, 19] by mechanisms still not well established. The prevalence of traditional CV risk factors does not seem to be higher in SSc [20–22], although the use of medications such as NSAID and oral glucocorticoids seems to contribute, at least in part, to this increased risk [9]. Recent clinical studies have shown a strong inverse correlation between CEC and CV disease prevalence [3] and incidence [23] in the general population. In our study, we found a significant decrease of CEC in SSc patients compared to controls after adjusting for confounders. Considering this relationship between reduced CEC and CV risk, it is reasonable to infer that CEC reduction may contribute to the increased atherosclerotic burden suffered by SSc patients [9, 19].

This is the first study to investigate CEC in SSc patients. However, it has been assessed in other connective tissue diseases like RA and SLE. Compared to RA patients, SSc patients appear to share a similar burden of subclinical atherosclerosis and cardiovascular comorbidities [24, 25]. In a previous report by our group including 178 RA patients and 223 sex-matched control subjects, CEC was not significantly different between them, although patients exhibiting higher disease activity had lower levels of CEC than patients in remission. Moreover, greater CEC was independently associated with a lower risk for the presence of carotid plaques in patients with RA [6]. Similarly, Ronda et al. evaluated CEC in 30 SLE patients and 30 healthy controls [5]. Although SLE was under control in most patients, CEC in patients was lower compared to that in controls. In a recent study by our group [26], CEC was impaired in SLE patients independently of other inflammation-related lipid profile modifications that occurred during the disease. Moreover, CEC was associated with carotid plaques in SLE patients. Based on the results in other inflammatory diseases, our findings on CEC are in line with the argument that inflammation affects CEC by mechanisms that are only partially understood.

The lack of association between traditional CV risk factors or lipid profiles with CEC in both SSc patients and controls observed in our study agrees with previous reports. In this sense, traditional risk factors are thought to explain only 3% of the variance observed in CEC [4]. Moreover, CEC cannot be explained by HDL cholesterol or apolipoprotein A1 levels [27]. Similarly, we did not find any association between statins and CEC in SSc patients and controls. This fact supports the claim that statins most likely confer their therapeutic benefits by means of a mechanism different from that promoting cholesterol efflux [3, 28].

The mRSS, a semiquantitative assessment of the extent of total skin sclerosis, has been used as a surrogate for disease activity, severity, and mortality in patients with SSc. The mRSS meets the filters of truth, discrimination, and feasibility [29]. No instrument, neither skin ultrasonography nor durometer, has outperformed the mRSS in clinical practice. The mRSS has also been proven to have prognostic value. In one study of 134 patients with diffuse skin involvement, a skin score of 20 or more was the third most powerful predictor of mortality after cardiac and pulmonary involvement [30]. A skin score of 20 or more was also the second most powerful predictor for the development of scleroderma renal crisis. Moreover, skin thickening is associated with improved survival and may therefore be useful as a surrogate for improved survival in clinical trials [29]. In our work, mRSS ranged from a minimum of 0 to a maximum of 27, with a median of 3 and an IQR between 1 and 6, indicating relatively low skin activity. However, it must be recalled that two thirds of the patients recruited had limited forms of SSc in which lower skin scores are expected to be found. We believe that if the mRSS had been higher, the relationship between the CEC and this score would probably have been stronger. Nevertheless, this association was significant. In our study, mRSS was univariably associated with age, hypertension, apolipoprotein B serum levels, and CEC. Remarkably, mRSS was still inversely associated with CEC when multivariable analysis was performed after adjusting for these variables. The fact that not only was CEC downregulated in patients with SSc, but also that skin thickness was responsible for this reduction in CEC, reinforces the findings of our study.

An abnormal body composition, a lower body mineral density, and sarcopenia have been found in patients with SSc [31, 32]. In our work, BMI and the presence of obesity were not different between groups. However, waist circumference was significantly higher in patients compared to controls (size effect 5 cm). We acknowledge that this limitation could have affected our results since the lipid profile or CEC could have varied depending on the body composition. For this reason, differences in CEC between populations were adjusted for waist circumference. Moreover, no relation was found between mRSS and BMI or abdominal circumference. Thus, no adjustment for this variable was necessary in our analysis of the relation of mRSS to CEC. For all of these reasons, we believe that the association between mRSS and CEC found in our study was not confounded by the abnormal body composition that patients with SSC may have.

We acknowledge some additional limitations in our study. There are other methods of assessing cholesterol efflux in vitro. However, most research done in population-based cohorts has been carried out using the same in vitro assay as that described in our study. Although monocyte/macrophage populations in SSc may have a specific phenotype that could affect CEC, it is not known how these phenotypes may affect CEC, not only in healthy population but also in inflammatory diseases. Additionally, we acknowledge that the assessment of cytokines or chemokines in our work would have been of interest since other inflammatory mechanisms are probably related to CEC changes. Other potential limitation is that controls were not matched for statins. However, as stated above, previous studies have demonstrated that statin therapy does not alter either CEC or the total mass of HDL cholesterol subclasses [3, 28]. Moreover, identical results have been found irrespective of matching or not matching when multivariable regression analysis was applied in epidemiological cross-sectional studies [33]. Because statins do not seem to affect CEC, we believe that the multivariable analysis performed in our study is capable of handling confounding situations including statin use.

## Conclusions

SSc patients show an abnormal lipid profile respect to control. In these patients, CEC is downregulated independently of other modifications to the lipid profile that occur in this autoimmune disease. Skin thickness, a known marker of SSc severity and damage, was associated with a lower CEC. Further studies are needed to assess whether this disruption in CEC is also related to the increased atherosclerotic risk commonly found in this disease.

## Supplementary Information

**Additional file 1: Supplementary Figure 1.** Multivariable analysis of the differences between scleroderma patients and controls in main lipid profile-related molecules (total cholesterol, triglycerides, HDL-cholesterol, apolipoprotein B and lipoprotein -mg/dl-, and cholesterol efflux capacity -%-).

**Additional file 2: Supplementary Table 1.** Differences in cardiovascular risk factors, lipid profiles and disease-related data between SS types.

## Data Availability

The datasets used and/or analyzed during the current study are available from the corresponding author, who has the ORCID identifier 0000-0002-4139-9295, on reasonable request.

## Abbreviations

ANA: Antinuclear antibodies
BMI: Body mass index
CEC: Cholesterol efflux capacity
cIMT: Carotid intima-media thickness
CRP: C-reactive protein
CV: Cardiovascular
DMARDs: Disease-modifying anti-rheumatic drugs
HDL: High-density lipoproteins
ILD: Interstitial lung disease
IQR: Interquartile range
LDL: Low-density lipoproteins
mRSS: Modified Rodnan skin score
RA: Rheumatoid arthritis
SD: Standard deviation
SLE: Systemic lupus erythematosus
SSc: Systemic sclerosis
