# Supplementary figures and images for: HDL cholesterol efflux capacity and lipid profile in patients with systemic sclerosis
Source: Arthritis Res Ther. 2021 Feb 23;23:62. doi: 10.1186/s13075-021-02443-9 (PMC7901093; doi:10.1186/s13075-021-02443-9)

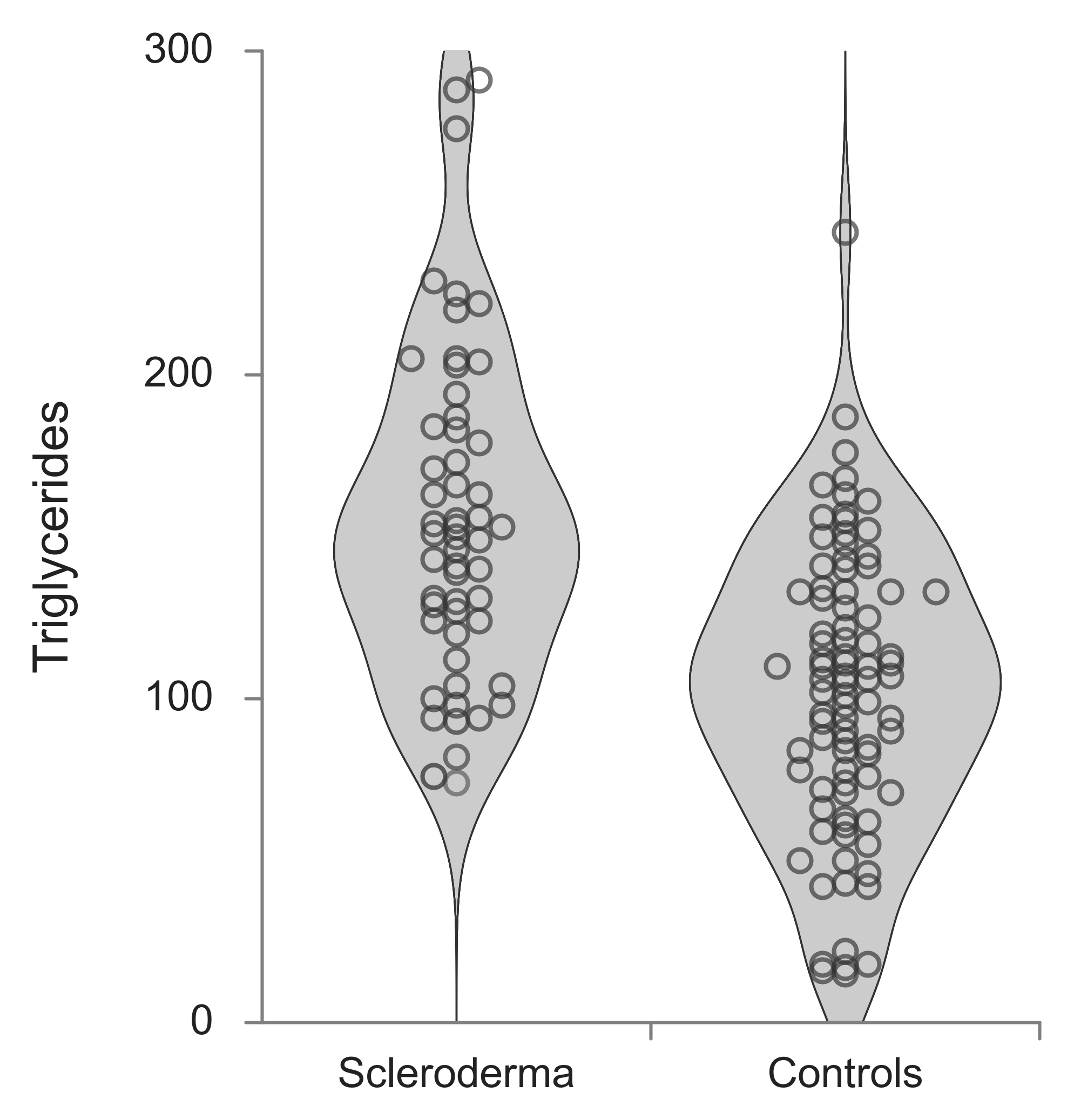

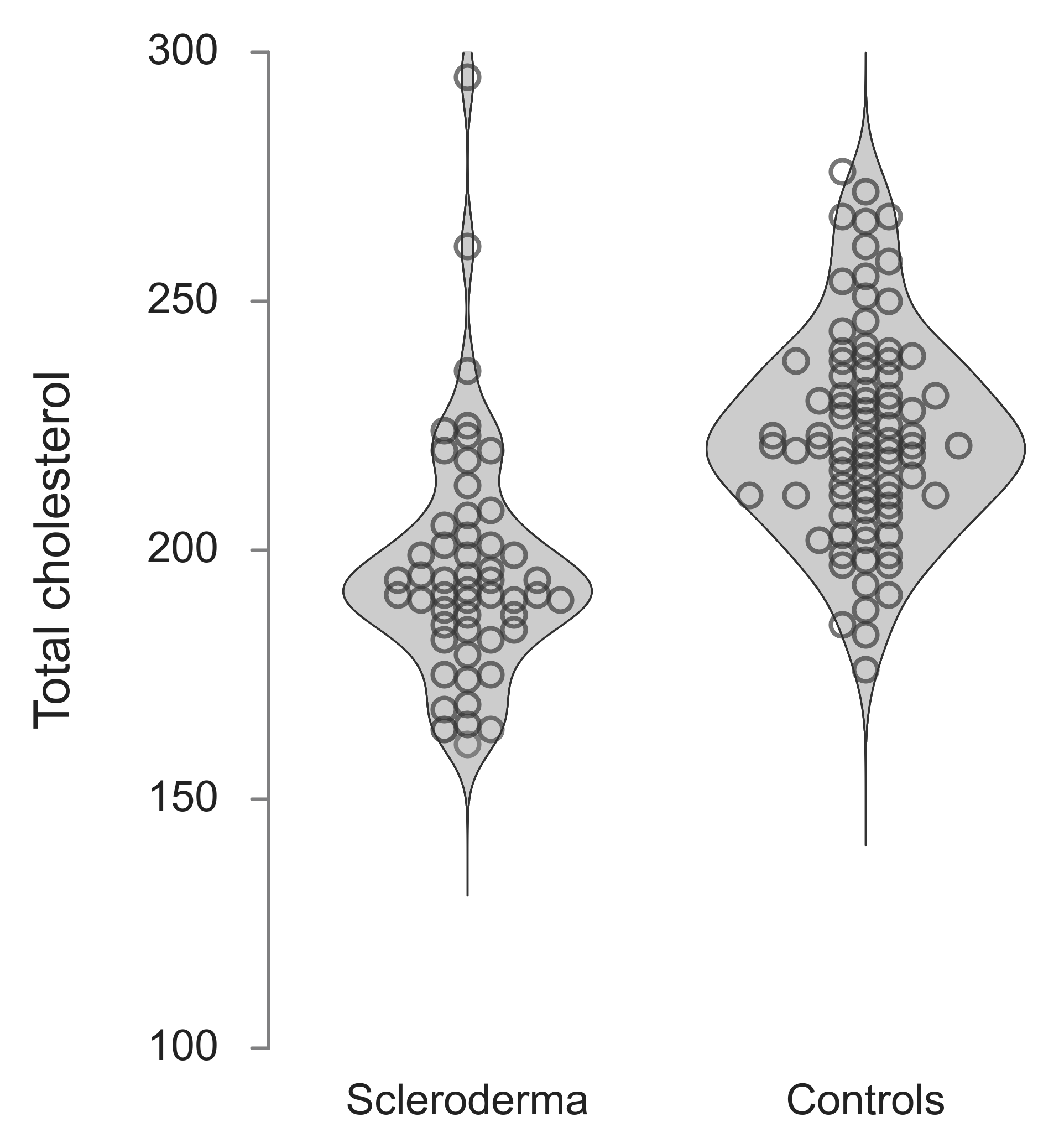
 **Supplementary Figure 1**

p=0.004

p=0.033


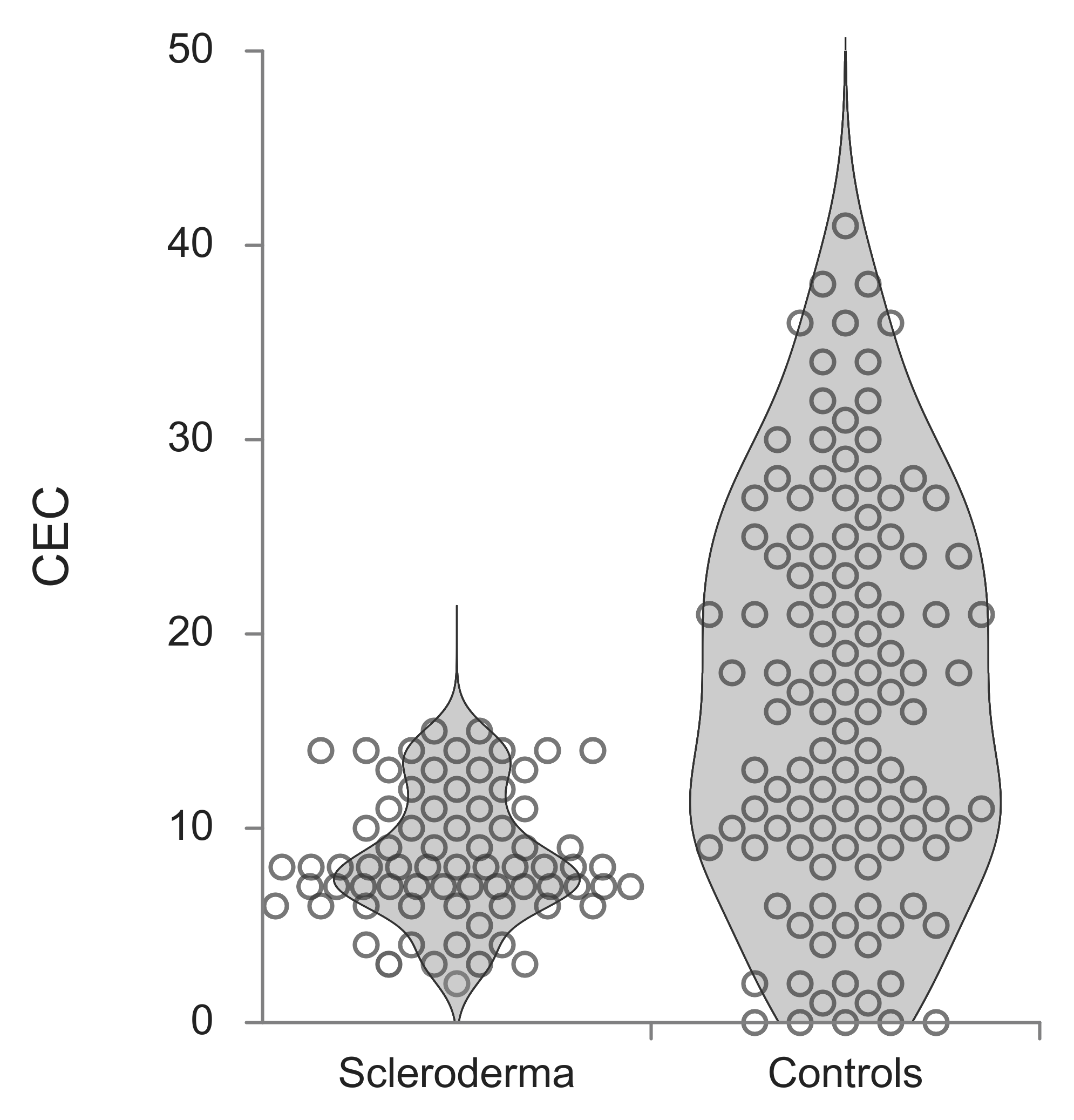

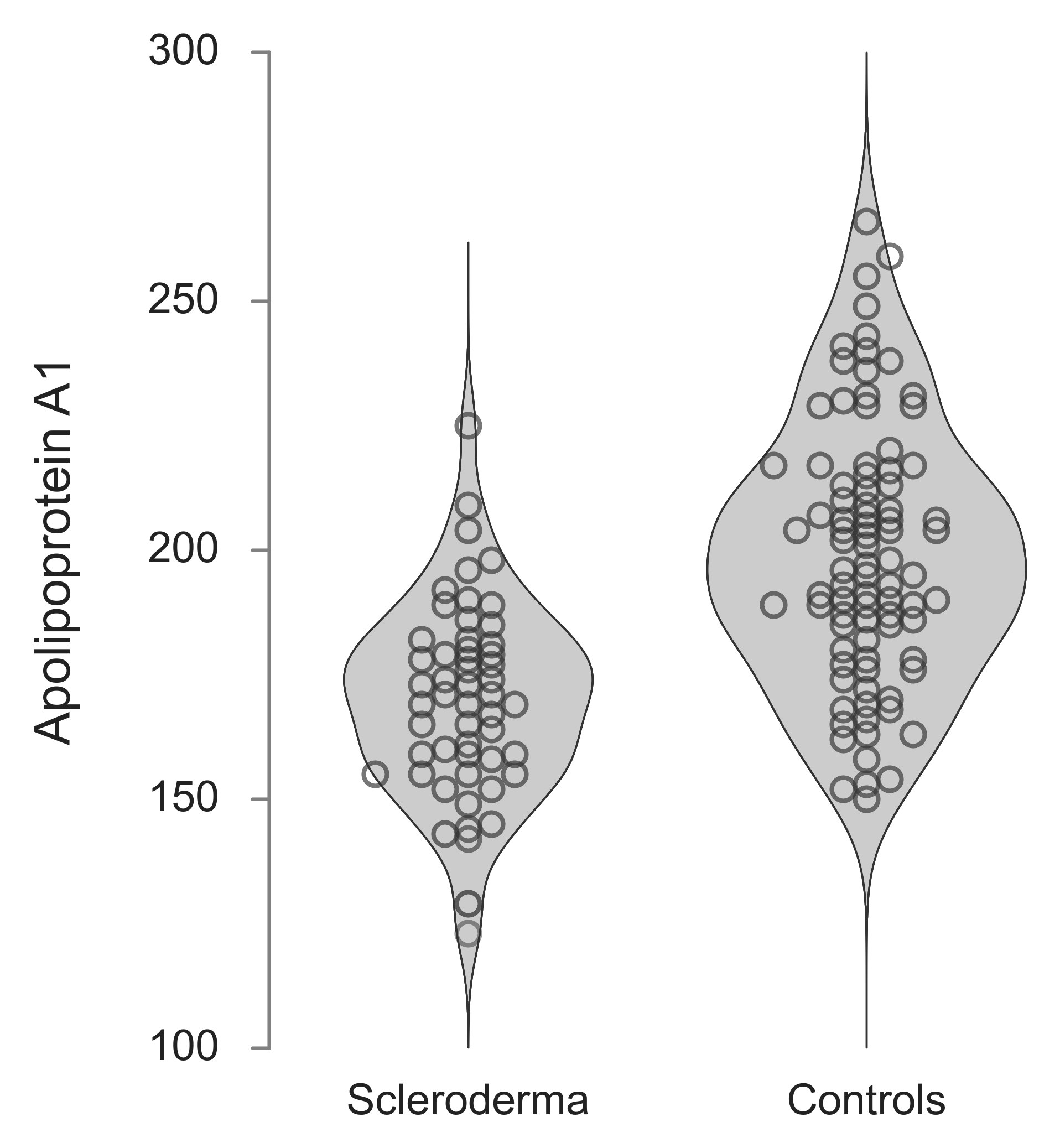

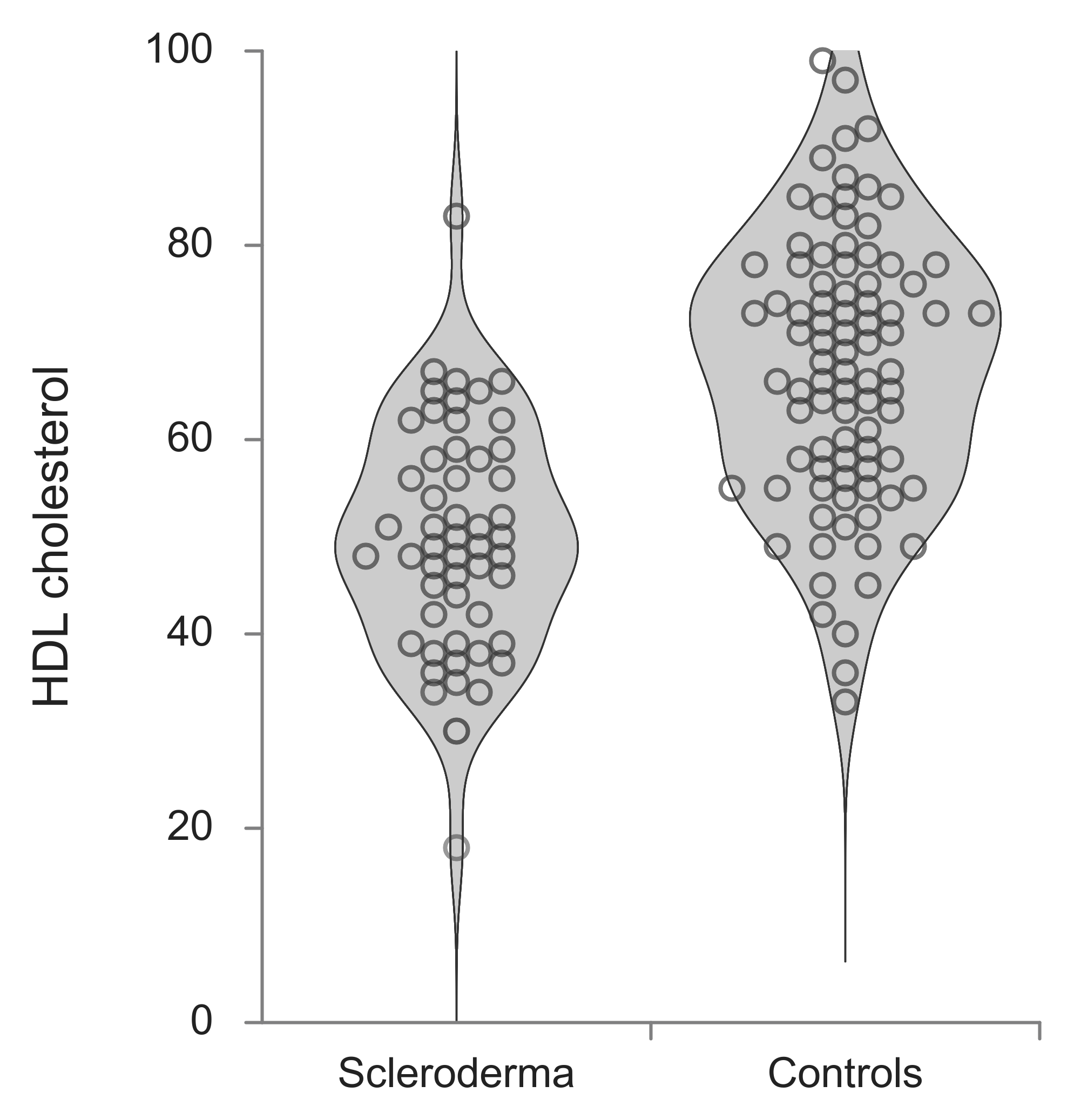

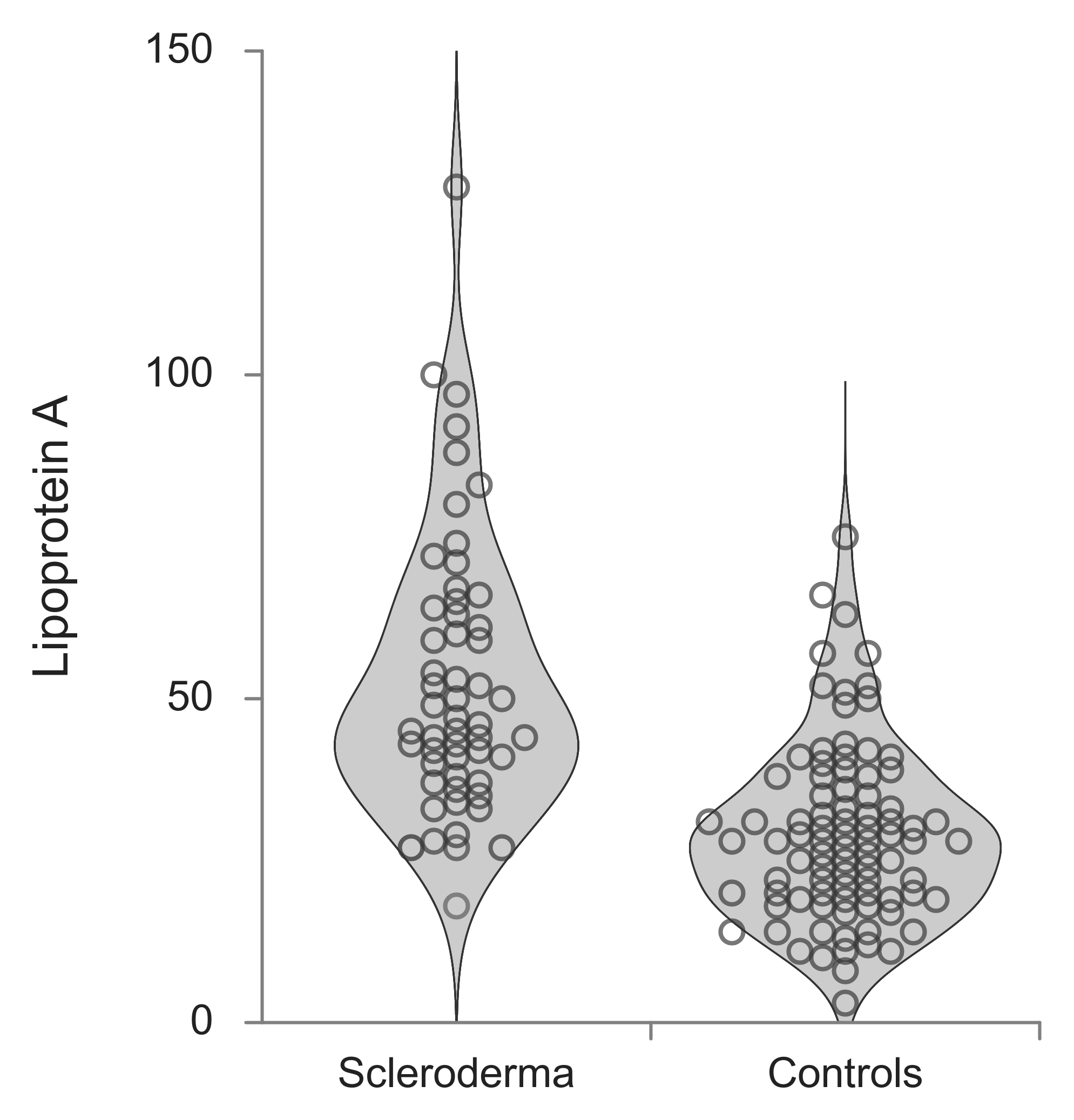


p=0.75

p=0.062

p=0.002

p=0.033

Supplement: Supplementary file 1 — Additional file 1: Supplementary Figure 1. Multivariable analysis of the differences between scleroderma patients and controls in main lipid profile-related molecules (total cholesterol, triglycerides, HDL-cholesterol, apolipoprotein B and lipoprotein -mg/dl-, and cholesterol efflux capacity -%-). [file 13075_2021_2443_MOESM1_ESM.docx]
