# Supplementary material for: HDL cholesterol efflux capacity and lipid profile in patients with systemic sclerosis
Source: Arthritis Res Ther. 2021 Feb 23;23:62. doi: 10.1186/s13075-021-02443-9 (PMC7901093; doi:10.1186/s13075-021-02443-9)
Supplement: Supplementary file 2 — Additional file 2: Supplementary Table 1. Differences in cardiovascular risk factors, lipid profiles and disease-related data between SS types. [file 13075_2021_2443_MOESM2_ESM.docx]

| **Supplementary Table 1. Differences in cardiovascular risk factors, lipid profiles and disease-related data between SS types.** | | | | | |
| --- | --- | --- | --- | --- | --- |
|  |  | SSc type | |  |  |
|  |  | Limited (n=50) | Diffuse (n=23) |  |  |
| Demographics | |  |  | p | p Adjusted* |
|  | Female, n (%) | 46 (92) | 22 (96) | 0.98 | - |
|  | Age, years | 58 ± 11 | 61 ± 10 | 0.28 | - |
|  | BMI, mg/cm2 | 28 ± 6 | 29 ± 5 | 0.42 | - |
|  | Waist circumference, cm | 93 ± 14 | 101 ± 13 | **0.027** | **-** |
|  | Systolic pressure, mmHg | 133 ± 20 | 133 ± 17 | 0.98 | - |
|  | Diastolic pressure, mmHg | 80 ± 10 | 81 ± 17 | 0.75 | - |
| Comorbidities | |  |  |  |  |
|  | Hypertension, n (%) | 15 (30) | 12 (52) | 0.068 | - |
|  | Current smoking, n (%) | 10 (20) | 4 (17) | 0.99 | - |
|  | Diabetes, n (%) | 3 (6) | 5 (22) | 0.99 | - |
|  | BMI > 30, n (%) | 14 (28) | 8 (35) | 0.56 | - |
|  | Statins, n (%) | 13 (26) | 12 (52) | **0.033** | **-** |
| Analytical data | |  |  |  | - |
|  | CRP, mg/dl | 2.16 (1.16-3.47) | 3.32 (1.06-5.27) | 0.16 | - |
|  | Cholesterol, mg/dl | 201 ± 38 | 193 ± 42 | 0.43 | - |
|  | Triglycerides, mg/dl | 143 ± 70 | 174 ± 104 | 0.18 | 0.96 |
|  | HDL-cholesterol, mg/dl | 49 ± 14 | 49 ± 10 | 0.94 | - |
|  | LDL-cholesterol, mg/dl | 115 ± 48 | 102,96 | 0.32 | - |
|  | LDL:HDL cholesterol ratio | 2.64 ± 1.81 | 2.21 ± 1.12 | 0.34 | - |
|  | Non-HDL cholesterol, mg/dl | 152 ± 40 | 143 ± 45 | 0.44 | - |
|  | Lipoprotein A, mg/dl | 19 (14-46) | 29 (8-130) | 0.22 | - |
|  | Apolipoprotein A1, mg/dl | 162 ± 33 | 181 ± 29 | **0.038** | 0.18 |
|  | Apolipoprotein B, mg/dl | 103 ± 27 | 94 ± 24 | 0.28 | - |
|  | Apo B: Apo A ratio | 0.68 ± 0.34 | 0.53 ± 0.14 | 0.28 | - |
|  | Atherogenic index | 4.50 ± 1.86 | 4.10 ± 1.46 | 0.42 | - |
|  | Cholesterol efflux capacity, % | 7.98 ± 3.22 | 9.79 ± 3.52 | **0.035** | 0.18 |
| SSc-related data | |  |  |  | - |
|  | Disease duration, years | 8 (4-15) | 12 (6-17) | 0.13 | - |
|  | Modified Rodnan Skin Score, units | 3 (0-6) | 5 (2-13) | 0.12 | - |
|  | Raynaud phenomenon, n (%) | 7 (14) | 4 (17) | 0.49 | - |
|  | Digital ulcers, n (%) | 6 (12) | 2 (9) | 0.99 | - |
|  | Calcinosis, n (%) | 6 (12) | 1 (4) | 0.42 | - |
|  | Arthritis, n (%) | 7 (14) | 8 (35) | **0.041** | **-** |
|  | Gastric reflux, n (%) | 19 (38) | 10 (43) | 0.70 | - |
|  | Pathological esophageal manometry, n (%) | 29 (58) | 11 (48) | 0.75 | - |
|  | Interstitial lung disease, n (%) | 7 (14) | 9 (39) | **0.027** | **-** |
|  | Pulmonary hypertension, n (%) | 12 (24) | 4 (17) | 0.54 | - |
|  | Anti-centromere antibody, n (%) | 41 (82) | 6 (26) | **0.000** | **-** |
|  | Anti-Scl70 antibody, n (%) | 2 (4) | 8 (35) | **0.001** | **-** |
| Treatments | |  |  |  |  |
|  | Current prednisone, n (%) | 2 (4) | 8 (35) | 0.43 | - |
|  | Prednisone, mg/day | 5 (5-7.5) | 5 (5-10) | 0.70 | - |
|  | DMARDs, n (%) | 3 (6) | 7 (30) | **0.009** | **-** |
|  | Methotrexate, n (%) | 0 (0) | 2 (9) | 0.096 | - |
|  | Hydroxychloroquine, n (%) | 2 (4) | 0 (0) | 0.99 | - |
| Data represent means±SD or median (IQR) when data were not normally distributed. | | | | | |
| BMI: body mass index; CRP: C-reactive protein; LDL: low-density lipoprotein. DMARD: disease-modifying antirheumatic drug. HDL: high-density lipoprotein. | | | | | |
| *Differences in lipid profile were adjusted for those variables covering cardiovascular comorbidities (waist circumference, statins intake, CRP and hypertension) with p values inferior to 0.20 in the comparison between SSc types. | | | | | |
